# The Association of the Phylogenetic Typing of the *Klebsiella pneumoniae* Isolates with Antibiotic Resistance

**DOI:** 10.1155/2021/1316992

**Published:** 2021-11-05

**Authors:** Shabnam Baghbanijavid, Hossein Samadi Kafil, Safar Farajniya, Seyed Reza Moaddab, Hasan Hosainzadegan, Fatemeh Yeganeh Sefidan, Mojtaba Varshouchi, Hamed Ebrahimzadeh Leylabadlo, Reza Ghotaslou

**Affiliations:** ^1^Infectious and Tropical Diseases Research Center, Tabriz University of Medical Sciences, Tabriz, Iran; ^2^Department of Microbiology, Faculty of Medicine, Tabriz University of Medical Sciences, Tabriz, Iran; ^3^Student Research Center, Tabriz University of Medical Sciences, Tabriz, Iran; ^4^Drug Applied Research Center, Tabriz University of Medical Sciences, Tabriz, Iran; ^5^Department of Laboratory Sciences, Faculty of Paramedicine, Tabriz University of Medical Sciences, Tabriz, Iran; ^6^Department of Microbiology, Faculty of Medicine, Maragheh University of Medical Sciences, Maragheh, Iran; ^7^Liver and Gastrointestinal Diseases Research Center, Tabriz University of Medical Sciences, Tabriz, Iran

## Abstract

*Klebsiella pneumoniae* complex (*KPC*) accounts for approximately one-third of all Gram-negative infections. Moreover, it is highly resistant and can taxonomically be distributed into KpI, KpII, and KpIII phylogroups. This study aimed to investigate the distribution of phylogenetic groups and the relationship between them and antibiotic resistance patterns. For this purpose, we collected *KPC* isolates from Tabriz, Iran, between 2018 and 2020. Antimicrobial susceptibility testing was performed by disk diffusion agar, and phylogenetic groups were then examined using *gyrA* restriction fragment length polymorphism (RFLP) and *parC* PCR methods. A total of 100 *KPC* isolates were obtained from the clinical specimens (urine, respiratory secretion, blood, wounds, and trachea). The enrolled patients included 47 men and 53 women aged from 1 to 91 years old. The highest sensitivity was found related to fosfomycin as 85%, followed by amikacin as 66%. The three phylogenetically groups by the RFLP-PCR method were found in *KPC*, 96% (96 isolates) as KpI, 3% (3 isolates) as KpII, and 1% (1isolate) as KpIII. The highest antibiotic resistance was observed in KpI. It was shown that a valid identification of three phylogenetic groups of *KPC* can be done by combining both *gyrA* PCR-RFLP and *parC* PCR. Of note, the KpI group was also observed as the dominant phylogenetic group with the highest resistance to antibiotics.

## 1. Introduction

Edwin Klebs first described *Klebsiella pneumoniae* complex (*KPC*) organisms in 1875, while studying the airways of patients who died due to pneumonia [1]. Additionally, Carl Friedlander formally described the species in 1882 [1, 2]. *KPC* is a Gram-negative bacterium from the *Enterobacteriaceae* family that can consequently cause various types of infections in human beings and is also found in both animals and plants [2]. *KPC* has a large adjunct genome of plasmids and chromosomal gene loci. In addition, *KPC* strains can be divided into multidrug-resistant, hypervirulent, and opportunistic groups based on the accessory genome [3]. Unfortunately, no approved potential therapy currently exists for some hypervirulent strains of this organism. Therefore, *KPC* can be classified as ESKAPE organisms [4], which “is a group consisting of the six most important microorganisms resistant to antimicrobials worldwide (*Enterococcus faecium*, *Staphylococcus aureus*, *KPC, Acinetobacter baumannii*, *Pseudomonas aeruginosa*, and *Enterobacter* spp.)” [5]. Of note, the important virulence factors of *KPC* were indicated to be resistant to serum, lipopolysaccharide, siderophores, pili, capsular antigens, and adhesions [6]. This bacterium is responsible for approximately one-third of all Gram-negative infections such as surgical wound infections, cystitis, urinary tract infections, pneumonia, endocarditis, and sepsis [7]. Moreover, it is often found in inflammation of necrotic lungs, pyogenic liver abscesses, internal endophthalmitis, meningitis, necrotizing fasciitis, and splenic abscesses [8]. *KPC* limits the treatment options for several groups of antibiotics by acquiring antibiotic resistance and innate resistance genes [9]. Currently, *KPC* strains by producing various types of *β*-lactamases (ESBLs) and carbapenemases can be found almost in all around the world [9]. *KPC* is classically and taxonomically divided into the KpI, KpII, and KpIII phylogroups [9, 10]. Recently, it has been suggested that these phylogroups, which are collectively known as the *KPC*, can be classified as separate species as follows: *K*. *pneumoniae* (KpI), *K*. *quasipneumoniae* (KpII), and *K*. *variicola* (KpIII) [11]. Up to now, several methods such as restriction fragment length polymorphism (RFLP-PCR), phylogenetic analysis of the *ori* region [12], phylogenetic analysis by sequencing the *dnaJ* gene [13], *gyrB* gene sequence, and the *parC* gene [14] have been introduced in order to determine the phylogenetic type of *Enterobacteriaceae*. Sequence analyses of *gyrA* and *parC* genes have shown that these genes are the reliable phylogenetic markers because they are not prone to repeated horizontal translocation across clusters [14]. Therefore, this study aimed to investigate the distribution of phylogenetic groups in *KPC* obtained from Tabriz city as well as determining the relationship between antibiotic resistance patterns and phylogenetic groups using the RFLP-PCR method.

## 2. Materials and Methods

### 2.1. Bacterial Isolation and Identification

At this stage, a total of 100 *KPC* isolates were obtained from various clinical specimens (including urine, respiratory secretion, blood, wounds, and trachea) at Imam Reza Hospital, Tabriz, Iran, between 2018 and 2020. Thereafter, the organisms were identified by the microscopic feature and differential tests [15]. Finally, these were stored in the tryptic soy broth medium containing glycerol and preserved at −70°C [16]. Notably, informed consent was obtained from all the adult participants and their parents or from legal guardians of minors participating in this study.

### 2.2. Antibiotic Susceptibilities

At this stage, the susceptibility testing was performed using the modified Kirby–Bauer method in Mueller-Hinton Agar Merck Co. (Darmstadt, Germany) for classes of antibiotics (Mast, Merseyside, UK), including fluoroquinolones (ciprofloxacin (CIP, 5 *μ*g)), aminoglycoside (amikacin (AK, 30 *μ*g) and gentamicin (GEN, 10 *μ*g)), beta-lactams (ampicillin (AMP, 10 *μ*g), imipenem (IPM, 10 *μ*g), ertapenem (ERT, 10 *μ*g), ceftazidime (CAZ, 30 *μ*g), cefepime (FEP, 30 *μ*g), ceftriaxone (CRO, 30 *μ*g), piperacillin (PIP, 100 *μ*g), and piperacillin/tazobactam (PTZ, 100/12.5 *μ*g)), antimetabolites (co-trimoxazole (SX, 30 *μ*g)), and fosfomycin (FOS, 200/50 *μ*g), which were interpreted in terms of the guidelines of the Clinical and Laboratory Standards Institute (CLSI) [17]. Moreover, in the current study, *K*. *pneumoniae* ATCC 12022 was used as the quality control strain.

### 2.3. DNA Preparation and Identification by the RFLP-PCR of *gyrA* Gene

The DNA extraction process was performed using the standard cell lysis. The isolates were then confirmed by a 441-bp PCR fragment of the *gyrA* gene (5`′-CGCGTACTATACGCCATGAACGTA-3′) and *gyrA*-C (5′-ACCGTTGATCACTTCGGTCAGG-3′) [14]. Thereafter, the RFLP-PCR was performed using restriction enzymes TaqI and HaeIII [18]. Thereafter, in order to confirm the identification of the KpII group, PCR was performed using the *parC* primer (*parC*2-1 (5′-GGCGCAACCCTTCTCCTAT-3′) and *parC*2-3 (5′-GAGCAGGATGTTTGGCAGG-3′)), which is specific to the KpII genotype, a 232 bp PCR product of which is only sound in the KpII isolates [19]. Finally, the RFLP products underwent electrophoresis in 2.5% agarose gel, followed by staining with 0.5 *μ*g/mL safe stain and visualizing under ultraviolet light.

### 2.4. Statistical Analysis

The results were analyzed using the descriptive statistics in SPSS software for Windows (Version 23 SPSS Inc., Chicago, IL, USA). *P* values ≤0.05 were considered as statistically significant.

## 3. Results

In this study, based on the statistical analyses, a total of 100 *KPC* were collected from 47 (47%) male and 53 (53%) female cases. The mean age of the included patients (47 men and 53 women) was 25 ± 41 years old. The sources of the collected samples were urine (58%), blood (16%), wounds (9%), trachea (9%), and respiratory secretion (8%). Notably, the obtained samples were isolated from different wards, including internal (27%), ICU (22%), infectious (18%), surgery (13%), pediatric (11%), and emergency (9%) wards.

**Figure 1:**
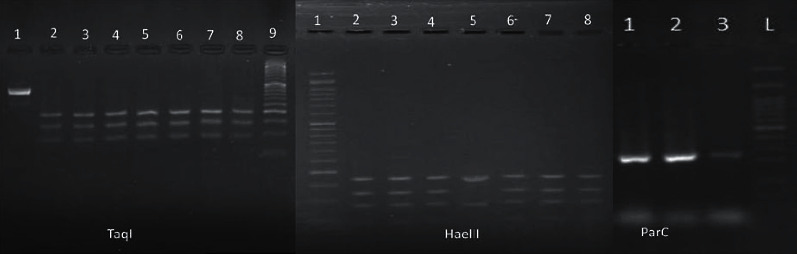
Taql, electrophoresis of the *GyrA* gene after adding the TaqI enzyme. Lane 1, electrophoresis of the *GyrA* gene prior to adding the enzyme with 441 bp bandwidth; lanes 2, 3, and 4, KpI group; lane 5, KpIII group; lanes 6, 7, and 8, KpII group; lane 9, 50 bp DNA ladder. HaeIII, electrophoresis of the *GyrA* gene after adding the HaeIII enzyme. Lane 1, 50 bp DNA ladder; lanes 2, 3, and 4, KpI group; lane 5, KpIII group; lanes 6, 7, and 8, KpII group. *parC*, *parC* gene electrophoresis to confirm the KpII group with 232 bp bandwidth. Lanes 1, 2, and 3, *parC* positive isolates with 232 bp bandwidth; lane 4, 100 bp DNA ladder. In KpI strains, the Taql enzyme created four bonds (including 197 bp, 142 bp, 93 bp, and 9 bp) and the HaeIII enzyme created four bonds (including 175 bp, 129 bp, 92 bp, and 45 bp). In KpII strains, the Taql enzyme created three bonds (including 197 bp, 151 bp, and 93 bp) and the HaeIII enzyme created four bonds (including 175 bp, 129 bp, 92 bp, and 45 bp). In KpIII strains, the Taql enzyme created four bonds (including 197 bp, 142 bp, 93 bp, and 9 bp) and the HaeIII enzyme created three bonds (including 175 bp, 174 bp, and 92 bp).

Table 1 provides the susceptibility testing of the *KPC* isolates to different antibiotics. The phylogenetic typing of the clinical *KPC* isolates was successfully done using the RFLP-PCR method. In the present study, 96 isolates were identified as KpI, 3 isolates as KpII, and only 1 isolate as KpIII (Figure 1).

The number of bacteria (five strains of KpI, three strains of KpII, and one strain of KpIII) was confirmed by sequence analysis of both *gyrA* and *parC* genes. The highest percentage of antibiotic resistance was observed in the KpI group, followed by the KpII and KpIII groups (*P* value ≤0.05), respectively (Table 1). There was a significant statistical difference in the *KPC* groups in terms of resistance to antibiotics and wards (*P* values ≤0.05) (Tables 1 and 2).

## 4. Discussion

*KPC* is responsible for both nosocomial and community infections, and the resistance to antibiotics among *KPC* is very important in this regard. Correspondingly, KpI, KpII, and KpIII were found in 96 isolates, three isolates, and one isolate, respectively. The phylogenetic types have also been found to be associated with drug resistance.

Brisse et al. in their study have reported the frequency of group I as 82.1%, KpII as 6.9%, and KpIII as 11%. Additionally, resistance to all antibiotics was more common in KpI compared to the other types, and a significant relationship was also found between antibiotic resistances and both KpI and KpII [19]. Silva de Melo et al. in their study have analyzed phylogenetic groups *KPC* isolated from nosocomial infections, community-based infections, and natural microbiota. As a result, they found that KpI had the most dominant phylogeny, and the highest percentage of antibiotic resistance was observed in the KpI, KpII, and KpIII groups, respectively [10]. In a previous study performed in Iran by Pajand et al., the clonal relationship between _bla_NDM/OXA-48-producing strains and the phylogenetic type of *KPC* was examined, and as a result, 80.3% of the isolates belonged to KpI, 16.4% to KpII, and 2.5% to the KpIII. Furthermore, most of the isolates producing NDM-1 (81%) belonged to KpI and the rest of them belonged to KpII and KpIII [20]. In a study conducted by Brisse and van Duijkeren [18], the distributions of three phylogenetic groups of *KPC* were examined in clinical isolates obtained from animals. All multidrug-resistant isolates belonged either to the KpI or to KpII phylogenetic group, and a significant relationship was found between antibiotic resistances and KpI and KpII [18]. In 2010, Younes et al. have reported the prevalence of _bla_CTX-M-15 transmissible isolates of *K*. *oxytoca* in hospitals and communities in Scotland. Interestingly, the phylogenetic type of all 219 *KPC* was assigned to KpI [21]. Moreover, Atmani et al. in Algiers have studied the virulence factors' characteristics and genetic background of ESBL-producing *KPC* isolated from wastewater. Besides, by performing *gyrA* PCR-RFLP phylogenetic analysis, it was shown that almost all strains of wastewater, similar to our clinical strains, belong to the phylogenetic group KpI [22]. Correspondingly, this result also highlights the pathogenicity of wastewater strains, which was shown to have a significant relationship with KpI [22]. Although these results were not obtained from isolates from human beings, to the best of our knowledge, *KPC* is an enteric pathogen that can transfer from food, animals, and water to humans. In a study conducted by Pons et al. in Mozambique from 2004 to 2009, characterization of *KPC*-producing ESBLs causing bacteremia and urinary tract infection was investigated. Accordingly, they have shown that all isolates of *KPC* belong to the same phylogenetic group (KpI) by *gyrA* gene analysis, and a significant relationship was also found between the high prevalence of KpI isolates and antibiotic resistance [23]. Due to the health status of the host, geographic climatic conditions, dietary factors, the history of the use of antibiotics, and host genetic factors, the rate of the phylogenetic groups is different among various populations [24].

In silicon restriction analysis of *gyrA* sequences obtained previously, it was shown that *gyrA* RFLP-PCR assigns isolates reliably to one of the three *KPC* groups [14]. Brisse et al. have also reported that all their 100 studied isolates had a distinct *gyrA* RFLP-PCR profile, which then matched with the reference strains and both the *gyrA* and *parC* sequencing [19]. Nowadays, we can investigate type *KPC* using some molecular assays such as RFLP and direct sequencing. Besides, it should be noted that the use of the RFLP-PCR method is more cost-effective than sequencing [14].

In the current study, in terms of the modified Kirby–Bauer method, the highest resistance rates were found to be related to ceftazidime (88%) and piperacillin (84%), respectively. As well, the most effective antibiotic against *KPC* was fosfomycin (85%), followed by amikacin (66%) and imipenem (50%), respectively. Moreover, Jazayeri et al. in their study have examined the patterns of antibiotic resistance *KPC* in Semnan, Iran. Accordingly, _bla_SHV and _bla_TEM were observed in 46.8% and 33.5%, and the highest resistance belonged to ceftazidime and aztreonam, and the lowest one belonged to imipenem and cefepime [25]. In the current study, the highest rate of resistance was found to be related to ceftazidime followed by that of piperacillin. As well, the lowest resistance to antibiotics was discovered to belong to fosfomycin followed by amikacin. In 2019, Shahi et al. studied the patterns of antibiotic resistance of *KPC* strains isolated from different clinical specimens in the teaching hospitals of Tabriz. In this regard, of 104 strains of *KPC*, the highest antibiotic resistance was observed to be related to co-trimoxazole, and the lowest resistance belonged to colistin [26]. Fonseca et al. in their study have mentioned that *KPC* has three separate species, namely, *K*. *pneumoniae* (KpI), *K*. *quasipneumoniae* (KpII), and *K*. *variicola* (KpIII). The presence of genes such as _bla_*KPC* in *K. variicola* and *K*. *quasipneumoniae* strains as well as NDM-1 metallo-*β*-lactamase in *K*. *variicola* is more worrying because these genes confer resistance to many different *β*-lactam antibiotics. Of particular concern is the evidence of homologous recombination among these three species of *Klebsiella* [27]. From 2018 to 2020, various studies have been conducted on antibiotic susceptibility testing, showing a growing trend of antibiotic resistance [28–30].

Since strains of *KPC* cause various infections, it has different virulence factors, pathogenicity, and resistance to antibiotics. In addition, infections caused by *KPC* are mostly associated with high mortality, long-term hospitalization, and high costs [7], so phylogenotyping is recommended in this regard. Accordingly, to ensure proper treatment, having understanding on the phylogenetic groups, how to use the drugs correctly, and how it can help to prevent drug resistance and reduce treatment costs, phylogenetic typing of *KPC* may be essential.

The *KPC* has three separate species, including *K*. *pneumoniae* (KpI), *K*. *quasipneumoniae* (KpII), and *K*. *variicola* (KpIII). However, currently, there is no specific biochemical test that can detect these three different species in the usual clinical microbiology laboratories [31]. Therefore, the usual identification of *Klebsiella* species is still difficult, as a result of which it is now known as *K*. *pneumoniae*, and we need a strong marker to detect these species [31]. Considering that these species have distinct pathogenic and epidemiological characteristics, controlling *Klebsiella* infections could be improved by applying an effective method such as RFLP.

Despite our promising results, this study also had limitations such as the relatively small sample size, limitations in statistical analyses, the lack of the resistance genes study, the relationship among these resistance genes and three different genotypes, and confocal direct sequencing of all strains to further explore the types.

## 5. Conclusion

In conclusion, reliable identification of the three *KPC* phylogenetic groups can be performed by combining *gyrA* PCR-RFLP and *parC* PCR. As well, KpI was found as the most common phylogenetic type. Different distributions of these genotypes indicate their distinct pathogenicity and epidemiological characteristics, which may play a role in the control of *KPC* infections. In the present study, we found that the rate of *KPC* resistance to antimicrobial agents is high, and the highest percentage of antibiotic resistance belonged to KpI, KpII, and KpIII, respectively.

## Figures and Tables

**Table 1:** Proportions of *K*. *pneumoniae* susceptibility testing in the three phylogenetic groups.

	Sensitive			Resistant			Intermediate		
Antibiotics	KpI	KpII	KpIII	KpI	KpII	KpIII	KpI	KpII	KpIII
Ampicillin	0	0	0	96	3	1	0	0	0
Piperacillin	5	0	1	82	1	0	19	2	0
Piperacillin/tazoba	34	3	1	43	0	0	9	0	0
Imipenem	46	3	1	30	0	0	20	0	0
Ertapenem	42	2	1	33	1	0	19	2	0
Cefepime	19	1	1	62	1	0	15	1	0
Ciprofloxacin	26	2	1	56	1	0	14	0	0
Co-trimoxazole	12	1	0	75	2	0	9	0	1
Fosfomycin	83	2	0	6	1	1	7	0	0
Gentamicin	22	2	1	62	1	0	12	0	0
Amikacin	63	2	1	21	1	0	12	0	0
Ceftazidime	10	1	1	83	1	0	3	1	0
Ceftriaxone	8	2	1	80	1	0	8	0	0

**Table 2:** The association among gender, wards, and sources in the phylogenetic groups.

	KPI	KPII	KPIII	*P* value
Gender				
Male	46	1	0	0.50
Female	50	2	1	

Sample				
Urine	56	2	0	0.35
Blood	15	0	1	
Wound	9	0	0	
Trachea	9	0	0	
Sputum	7	1	0	

Wards				
Internal	27	0	0	0.03
ICU	21	1	0	
Infectious	18	0	0	
Surgery	13	0	0	
Emergency	9	0	0	
Pediatrics	8	2	1	

## Data Availability

The data used to support the findings of this study are available from the corresponding author upon request.
